# Trypanocide usage in the cattle belt of southwestern Uganda

**DOI:** 10.3389/fmicb.2023.1296522

**Published:** 2023-12-13

**Authors:** Keneth Iceland Kasozi, Ewan Thomas MacLeod, Keith Robert Sones, Susan Christina Welburn

**Affiliations:** ^1^Infection Medicine, College of Medicine and Veterinary Medicine, Biomedical Sciences: Edinburgh Medical School, College of Medicine and Veterinary Medicine, The University of Edinburgh, Edinburgh, Scotland, United Kingdom; ^2^School of Medicine, Kabale University, Kabale, Uganda; ^3^Keith Sones Associates, Warkworth House, Warkworth, Banbury, United Kingdom; ^4^Zhejiang University - University of Edinburgh Institute, Zhejiang University School of Medicine, Haining, China

**Keywords:** neglected tropical diseases, zoonoses, trypanocides, animal African trypanosomiasis, antimicrobial resistance (AMR), veterinary drug shops, pharmacovigilance, Stamp Out Sleeping Sickness

## Abstract

**Background:**

Systematic infrastructure and regulatory weaknesses over many decades, in communities struggling with animal African trypanosomiasis (AAT) would be expected to create an environment that would promote drug misuse and risk development of drug resistance. Here, we explore rural community practices of livestock keepers, livestock extension officers and drug shop attendants to determine whether appropriate practice was being followed in administration of trypanocides and other drugs.

**Methods:**

A questionnaire-based survey was undertaken in southwestern Uganda in 2022 involving 451 farmers who kept cattle, sheep or goats and 79 “professionals” who were either livestock extension officers or drug shop attendants.

**Results:**

Respondents reported using one or more type of trypanocidal drug on 80.1% of the 451 farms in the last 30 days. Diminazene aceturate was used on around three-quarters of farms, while isometamidium chloride was used on around one-fifth. Homidium bromide was used on less than 1% of farms. Cattle were significantly more likely to be treated with trypanocides than sheep or goats. On around two-thirds of farms, trypanocides were prepared and injected by farmers, with extension officers administering these drugs on most of the other third, especially on cattle farms. Almost all drugs were obtained from privately-owned drug shops. For treatment of AAT with trypanocides, prescription-only medicines were routinely used by farmers without professional supervision and in the absence of a definitive diagnosis. While a far greater proportion of professionals had a better education and had received training on the use of trypanocides than farmers, there was relatively little difference in their ability to use these drugs correctly. Farmers were more likely than professionals to use only DA to treat trypanosomiasis and were more likely to use antibiotics as well as trypanocidal drugs to treat the animal. Furthermore, they estimated, on average, that twice the recommended dose of either diminazene aceturate or isometamidium chloride was needed to treat a hypothetical 400 kg bovine. A minority of both farmers and professionals reported that they observed the recommended withdrawal times following injection of trypanocidal drugs and very few of either group knew the recommended withdrawal times for milk or meat. Only one in six farmers reported using the sanative pair (alternating use of diminazene aceturate and isometamidium chloride), to reduce the risk of drug resistant trypanosome strains emerging, while this approach was more widely used by professionals. Farmers reported using antibiotics more commonly than the professionals, especially in sheep and goats, raising concerns as to overuse and misuse of this critical class of drugs. In addition to using trypanocides, most farmers also reported using a topical veterinary pesticide for the control of ticks and tsetse. On average, farmers spent 12.2% of their income from livestock sales on trypanocides.

**Conclusion:**

This study highlights the complexity of issues involved in the fight against AAT using drug treatment. A multistakeholder campaign to increase awareness among farmers, drug shop attendants, and extension workers of the importance of adherence to recommended drug dosing, using the sanative pair and following recommended drug withdrawal guidance would promote best practice, reduce the risk of emergence of resistant strains of trypanosomes, and support enhanced food safety.

## 1 Introduction

Antimicrobial resistance (AMR) is considered by the World Health Organization (WHO) to be one of the top 10 global public health threats facing humanity (WHO, 2015, 2021). Best practice to minimize the risk of AMR is generally considered to include only using antimicrobials under the supervision of a fully qualified health professional on an individual patient or animal basis after a definitive diagnosis. Other features of best practice include using the right active ingredient at the right dose, administered in the correct way, respecting recommended withdrawal times, and not relying solely on a single active ingredient for prolonged periods of time. For vector-borne diseases, such as trypanosomiasis, integrated control which combines actions against the vector (tsetse) and pathogen (trypanosomes) is also recommended (FAO, 1998).

In the context of animal health on remote farms in low- and middle-income countries (LMICs), such as the cattle belt of southwestern Uganda where the current study was conducted, these ideals are especially hard to attain. Fully trained private veterinary professionals are often not present and even if they are, their services are not affordable for most livestock keepers. While government-employed district veterinary officers and livestock extension officers are usually present in these areas, they tend to be limited in their reach due to shortage of resources, especially inadequate transport, and are too few to reach all who could benefit from their services (Perry et al., 2005).

In such areas, alternative systems have emerged in which farmers have to be largely self-reliant and obtain their animal health products, advice and, in some cases, services almost entirely from privately-owned drug shops where they encounter staff ranging from untrained, through certificate and diploma holders to degree-educated professionals. Drug shop staff often have local knowledge, are community members (easy to access), cost effective and can balance their lack of technical training with understanding of cultural and traditional practices (Caudell et al., 2020).

While liberalization of animal health services has some associated benefits, such as increased access to medicines and potential economic growth, it has also generated enormous challenges including unregulated markets, misuse and overuse of veterinary products, regulatory weakness, and AMR, especially in countries where drug regulatory and distribution systems are weak (Jaime et al., 2022).

Trypanocide resistance in both humans (Kasozi et al., 2022) and animals (Wangwe et al., 2019; Kasozi et al., 2023) raises major public health risks following expression of cross-species resistance genes (Kasozi et al., 2022; Okello et al., 2022). Animal African trypanocide resistance has previously been associated with poor farming practices, farmer treatments, underdosing and untrained personnel (Ngumbi and Silayo, 2017; Kasozi et al., 2022).

In this study, we assessed farmer trypanocide (and other drug) usage practices in southwestern Uganda where antimicrobial drugs are heavily used.

## 2 Methods

**Figure 1:**
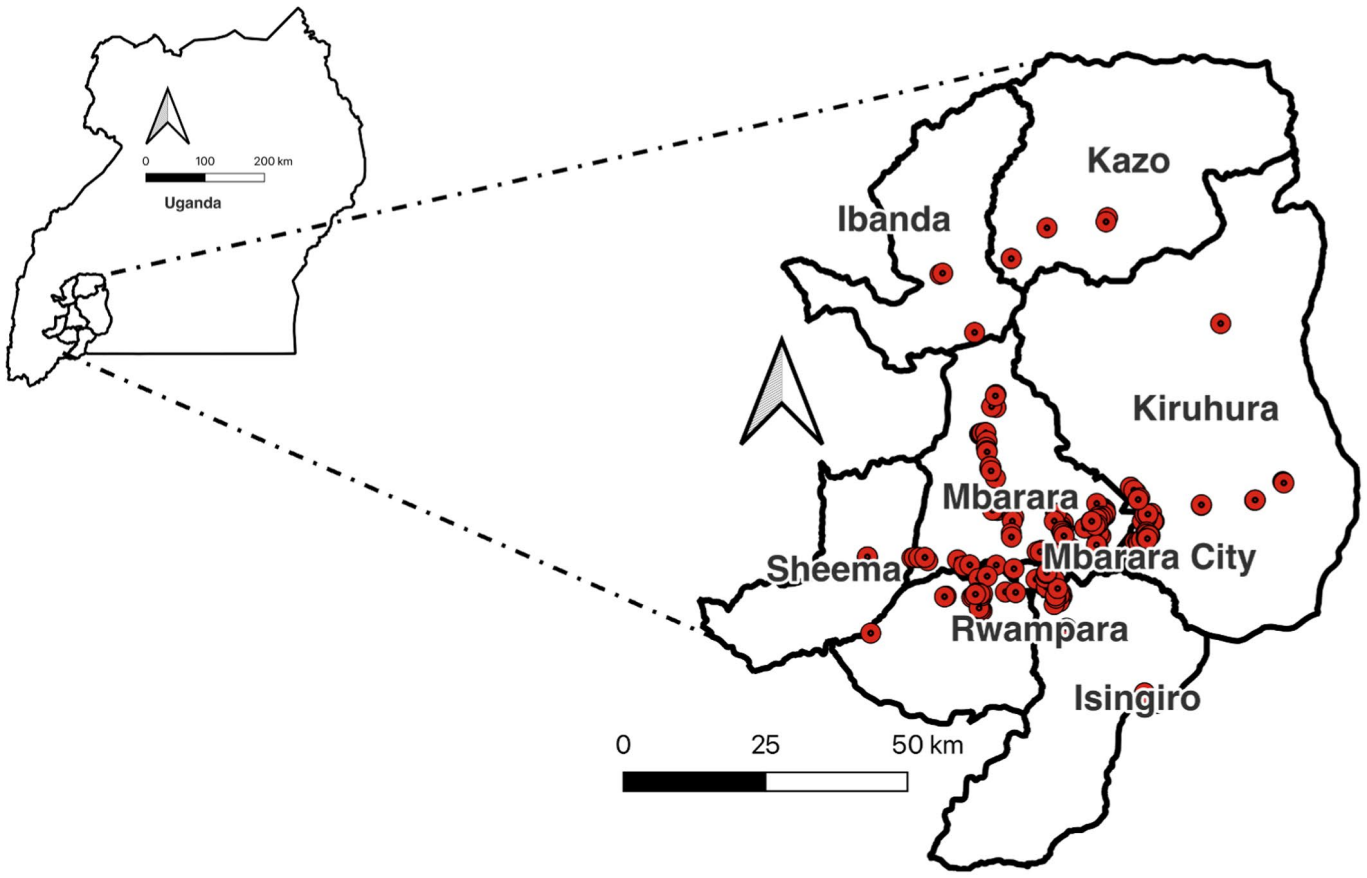
Survey villages in the seven districts and one city of southwestern Uganda along the cattle belt.

This study comprised of a questionnaire-based survey (Supplementary File 1) conducted in the cattle belt of southwestern Uganda between July and October 2022. The objective was to determine how trypanocidal drugs were being used in this area to control animal African trypanosomiasis in cattle, sheep, and goats. Focused on the districts of Ibanda, Isingiro, Kazo, Kiruura, Mbarara, Rwampara, and Sheema (Figure 1), the study team worked with district veterinary officers to identify livestock keeping communities in areas where trypanocide resistance was suspected. The study was approved by the Edinburgh Medical School Research Ethics Committee (22-EMREC-022) and in Uganda, it was approved by the Ethics Committee at the College of Veterinary Medicine of Makerere University (SVAR-IACUC/114/2022). After acquiring local consent from the respective chief administrative officers, 557 participants were purposefully recruited: 478 from farms and 79 who were district extension officers or drug shop attendants. Extension officers were persons working for the local government legally employed by the government, while drug shop attendants included assistants and technicians. Assistants are often relatives including wives, partners, and husbands of the owner of the drug shop by the National Drug Authority (NDA) standards. Technicians were individuals who held a veterinary certificate or diploma from one of the vocational institutions in Uganda and licensed to operate veterinary drug shops. The study protocol was registered with BMC ISRCTN (Supplementary File 2). The pre-tested questionnaire was written in English but administered in the appropriate local language by trained enumerators. Each questionnaire took around 30–45 min to complete. Responses to the questions in the questionnaire were recorded in English and entered directly into a tablet connected to the internet where possible; in areas without stable internet connection, responses were captured as hard copy and entered on a tablet as soon as possible. Respondents’ locations were recorded using a Global Positioning System (GPS), but these locations were anonymized and used only to illustrate their geographical distribution within the study site.

On farms, the respondents were whoever was available at the time of the visit and included members of the farming family as well as paid employees. District extension officers with responsibility for livestock who were included in the study were interviewed either at their official government offices or their private drug shops. In drug shops, respondents included both unqualified assistants and diploma-level technicians, who could be employees or owners (these two groups are referred to as “drug shop attendants” throughout this paper) as well as extension officers.

### 2.1 Statistical analysis

Survey responses were entered into Microsoft Office Forms which presented the data in MS Excel spreadsheets. This was exported into the open-source software R version 4.3.1 using the pacman, party, rio, and tidyverse packages which, in addition to MS Excel, were used to generate descriptive statistics. Odds ratios were calculated using the epiR package version 2.0.63; for observations with zero fields the odd ratios were generated using MedCalc® at 95% confidence interval. One sample tests were computed using the rstatix package and significance reported when *p < 0.05*. Since the focus of the study was use of trypanocidal drugs, which are primarily used in cattle, sheep, and goats, only farms that kept ruminants were included in the analyses (Supplementary File 3).

## 3 Results

### 3.1 Demographic information

The survey was administered to 478 farmers and 79 additional respondents who were drug shop attendants or extension officers (hereafter referred to as professionals). Twenty-seven farm respondents who reported that they did not keep ruminants were not included in the analyses making the farm sample size 451 (Table 1). These farmers were listed on a livestock keeping registry but did not hold any ruminant stock when they were visited.

**Table 1:** Number and percentages on demographic information for respondents.

Parameter	Variables	Farmers (*n* = 451)	Professionals (*n* = 79)
Sex	Female	111 (24.6)	36 (45.6)
	Male	340 (75.4)	43 (54.4)
Primary role	Farmer	306 (67.8)	
	Farm employee	145 (32.2)	
	Drug shop assistant		35 (44.3)
	Drug shop technician		28 (35.4)
	Extension worker		16 (20.3)
Education	No formal	200 (44.3)	2 (2.5)
	Basic	224 (49.7)	18 (22.8)
	Tertiary	27 (6.0)	59 (74.7)
Authority	Head	208 (46.1)	35 (44.3)
	Second-in-command	45 (10.0)	8 (10.1)
	Other family member	96 (21.3)	2 (2.5)
	Employee	102 (22.6)	34 (43.0)
Age (years)^*^	Children (14–17)	12 (2.7)	
	Young adults (18–25)	101 (22.5)	11 (13.9)
	Adults (26–35)	94 (20.9)	35 (44.3)
	Mature (36–45)	102 (22.7)	27 (34.2)
	Elderly (46–61)	105 (23.4)	6 (7.6)
	Most elderly (62–82)	35 (7.8)	
	Median (IQR)	37.0 (22.0)	35.0 (12.0)

^*^n = 449 for farmers as two participants withdrew consent.

The category “farmer” included farming heads of households, their spouses and other family members. The category “farm employee” were paid workers. On each farm only one person was interviewed so the 451 farmers represented 451 different farms. Around three-quarters (77.4%) of the farm respondents were farming family members and a little under a quarter (22.6%) were employees. Close to three-quarters (75.4%) of the farm respondents were male. Drug shop attendants and extension workers were more evenly split between men (54.4%) and women (45.6%). The age of farmers ranged from 14 to 82 years; just under 90% were in the range 18–61 years and the mean age was 38.8 (median = 37.0) years. Drug shop attendants and extension officers were all in the age range 18–61 years and their mean age was 35.2 (median = 35.0) years. A little over half of all respondents were the head or second-in-command of the farm or drug shop/extension office. Among farmers, most (94.0%) had received either no formal education or only basic education while among drug shop attendants and extension officers three-quarters (74.7%) had received tertiary education (Table 1).

Cattle were kept on 307 of the 451 farms (68.1%), with or without small ruminants. Both cattle and sheep/goats were kept on 191 farms (42.3%), cattle only on 110 (24.4%) and sheep/goats only on 150 (33.3%). More than half of respondents who kept cattle (51.0%) reported keeping crossbreeds, with the remainder relatively evenly split between exotic breeds (26.8%) and local breeds, most likely Ankole (22.1%). More than three-quarters of respondents (75.7%) who reported keeping small ruminants kept local breeds. Mean reported herd size for cattle was 104, although this covered a wide range from 1 to 20,000, with a median cattle herd size of 24. The mean flock size for small ruminants was 34, again with a wide range from 1 to 1,000 with a corresponding median flock size of 16. Respondents were often reluctant to report the exact number of animals they kept so these values should be treated with caution. On a little over half of farms (52.6%), other livestock species and/or domestic animals (dogs, cats, pigs, and chickens) were kept in addition to ruminants. Around two-thirds of respondents considered their farms to be semi-commercial with the remaining third classifying them as subsistence (Table 2).

**Table 2:** Livestock kept on respondents’ farms.

Types of livestock		Number (%) farms (*n* = 451)
Ruminant groups	Cattle only farms	110 (24.4)
	Sheep/goats only farms	150 (33.3)
	Both (ruminants)	191 (42.3)
	Cattle with/without sheep/goats	307 (68.1)
	Sheep/goats with/without cattle	348 (77.2)
Farm species	Ruminants only	214 (47.4)
	Ruminants + dogs/cats	92 (20.4)
	Ruminants + dogs/cats + pigs + chickens	82 (18.2)
	Ruminants + pigs + chickens	63 (14.0)
Ruminant breeds	Cattle crosses	152 (50.0)
	Exotic cattle	80 (26.1)
	Local (Ankole)	66 (21.0)
	Do not know (cattle)	9 (2.9)
	Median cattle herd size (IQR)	24 (39)
	Mean cattle herd size (range)	104 (1–20,000)
	Sheep/goat crosses	60 (17.2)
	Exotic sheep/goats	23 (6.7)
	Local sheep/goats	259 (75.4)
	Do not know (sheep/goats)	6 (1.7)
	Median sheep/goat herd size (IQR)	16 (26)
	Mean sheep/goat herd size (range)	34 (1–1,000)
Nature of farming	Semi-commercial	297 (65.9)
	Subsistence	154 (34.1)

### 3.2 Knowledge on trypanosomiasis

Among farmers, around a fifth (22.0%) knew that tsetse were involved in trypanosome transmission compared to more than half (53.2%) among professionals (Table 3). There was no statistically significant difference (*p* < 0.05) between the proportions of the two groups who were able to correctly identify either the best approach (i.e., trypanocide use against antibiotics, bush burning, ethnomedicine, and acaricide mono options) for the control of trypanosomiasis (64.3% of farmers compared to 68.3% of professionals selected “use of trypanocides” from a list of alternative but ineffective methods) or the season when the burden of the disease is highest (78.9% of farmers vs. 87.3% of professionals correctly selected “wet season”). While less than a quarter (23.9%) of farmers reported that they had received training on the correct use of trypanocides, more than four-fifths (87.3%) of professionals reported receiving such training (Table 3).

**Table 3:** Determinants of knowledge on trypanosomiasis based on extension meeting trainings, disease epidemiology and control in the study population.

Topic	Number (%) of correct farmer responses (*N* = 451)	Number (%) of correct professional responses (*N* = 79)	Odds ratio (95% CI)	Chi-square *p* values
Extension meetings trainings	217 (46.1)	66 (83.5)	5.5 (2.9–10.2)	<0.001
Trypanocides for disease control	290 (64.3)	54 (68.3)	1.2 (0.7–2.0)	0.57
Tsetse major vector	99 (22.0)	42 (53.2)	4.0 (2.4–6.6)	<0.0001
Disease in rainy season	356 (78.9)	69 (87.3)	1.8 (0.9–3.9)	0.08

### 3.3 Drug usage practices

Almost two-thirds (66.1%) of farmers reported that they prepared and injected trypanocidal drugs on their farms, with extension officers administering the drugs on 21.7% of farms and drug shop attendants administering trypanocides on 12.2% of farms. Drug shop attendants were significantly less likely to treat animals on farms that only kept cattle (7.3% of cattle only farms vs. 22.5% of sheep/goat only farms, *p* < 0.05). When asked about extension officers, 59.0% of farmers considered that they were accessible in their communities and 42.6% considered they were reliable with treatments (Table 4). Most respondents considered that private outlets were the cheapest source of trypanocidal drugs and nearly all drugs (97.3% of farm respondents) were reported to have been purchased from private drug shops.

**Table 4:** Major practice patterns in the study population.

Trypanocide practices		Farms with both cattle and sheep/goats (*N* = 191)	Sheep/goats only on farms (*N* = 150)	Farms with cattle but no sheep/goats (*N* = 110)	Farmers (*N* = 451)	Professionals (*N* = 79)	Farmers for sheep/goats vs. cattle		Farmers vs. Professionals	
							OR (95% CI)	*X*^2^-*p* value	OR (95% CI)	*X^2^*-*p* value
Administer treatments	Drug shop attendants	13 (6.8)	34 (22.7)	8 (7.3)	55 (12.2)	11 (13.9)	0.3 (0.1–0.6)	0.001	1.2 (0.6–2.3)	0.67
	Extension officer	44 (23.0)	25 (16.7)	29 (26.4)	98 (21.7)	16 (20.3)	1.8 (1.0–3.3)	0.06	0.9 (0.5–1.6)	0.77
	Farmers	134 (70.2)	91 (60.7)	73 (66.4)	298 (66.1)	52 (65.8)	1.3 (0.8–2.1)	0.35	1.0 (0.6–1.6)	0.96
Correct route	Avoid Intravenous	188 (98.4)	149 (99.3)	106 (96.4)	443 (98.2)	78 (98.7)	0.2 (0.0–1.4)	0.17*	1.4 (0.2–11.4)	1.00*
	Intramuscular	188 (98.4)	143 (95.3)	106 (96.4)	437 (96.9)	78 (98.7)	0.2 (0.0–1.5)	0.17*	2.5 (0.3–19.3)	0.71*
Lowest priced trypanocides	Private outlets	141 (73.8)	103 (68.7)	85 (77.3)	329 (72.9)	61 (77.2)	1.5 (0.8–2.7)	0.13	1.2 (0.7–2.2)	0.43
	Informal market	2 (1.0)	0 (0)	0 (0)	2 (0.4)	0 (0)	1.4 (0.0–69.2)	0.00*	1.1 (0.1–23.8)	1.00*
	Government	1 (0.5)	1 (0.7)	2 (1.8)	4 (0.9)	3 (3.8)	2.6 (0.2–81.9)	0.57	4.4 (0.8–21.7)	0.07*
	Uniform price	37 (19.4)	13 (8.7)	12 (10.9)	62 (13.7)	14 (17.7)	1.3 (0.6–3.0)	0.55	1.4 (0.7–2.6)	0.29
	Do not know	10 (5.2)	33 (22.0)	11 (10.0)	54 (12.0)	1 (1.3)	0.4 (0.2–0.8)	0.01	0.1 (0.0–0.5)	0.002*
Source of trypanocides	Private outlet	187 (97.9)	145 (96.7)	107 (97.3)	439 (97.3)	79 (100)	1.2 (0.3–6.4)	1.00*	4.5 (0.3–77.2)	0.23*
	Government	1 (0.5)	1 (0.7)	3 (2.7)	5 (1.1)	0 (0)	2.7 (0.3–80.6)	0.62*	0.5 (0.0–9.3)	1.00*
	Do not know	3 (1.6)	4 (2.7)	0 (0)	7 (1.6)	0 (0)	0.15 (0.0–2.8)	0.14*	0.4 (0.0–6.6)	0.60*
Water source	Borehole	15 (7.9)	5 (3.3)	8 (7.3)	28 (6.2)	4 (5.1)	2.2 (0.7–7.8)	0.17	0.8 (0.2–2.2)	1.00*
	Bottled	36 (18.8)	10 (6.7)	21 (19.1)	67 (14.9)	10 (12.7)	3.3 (1.5–7.6)	0.003	0.8 (0.4–1.6)	0.63
	Tap	53 (27.7)	61 (40.7)	45 (40.9)	159 (35.3)	34 (43.0)	1.0 (0.6–1.7)	0.97	1.4 (0.8–2.3)	0.19
	Stream	2 (1.0)	0 (0)	1 (0.9)	3 (0.7)	0 (0)	4.1 (0.2–102.1)	0.42*	0.8 (0.0–15.7)	1.00*
	Well	85 (44.5)	70 (46.7)	35 (31.8)	190 (42.1)	9 (11.4)	0.6 (0.3–0.9)	0.02	0.2 (0.1–0.4)	<0.0001
	Do not know	0 (0)	4 (2.7)	0 (0)	4 (0.9)	22 (27.8)	0.15 (0.0–2.8)	0.14*	41.3 (15.0–149.5)	<0.0001
Extension officer	Accessibility	116 (60.7)	78 (52.0)	72 (65.4)	266 (59.0)	71 (89.9)	0.6 (0.3–0.9)	0.03	6.2 (2.9–13.1)	<0.001
	Reliability	77 (40.3)	68 (45.3)	47 (42.7)	192 (42.6)	70 (88.6)	1.1 (0.7–1.8)	0.68	10.3 (5.3–22.7)	<0.0001

OR, Odds ratio. 95% CI, 95% confidence intervals with lower and upper limits reported. Superscript (*) indicates Fisher’s exact *p* value being reported.

Most of the farmers (96.9%) and professionals (98.7%) injected trypanocides using the recommended intramuscular route. Irrespective of who administered the trypanocidal drug, in all cases the water used to prepare the solution for injection came from a tap, bore hole, stream, or other non-sterile source; no respondents reported using commercially prepared water for injection (Table 4). Farmers on cattle only farms were almost three-times more likely to use bottled drinking water as farmers on sheep/goat only farms (19.1 vs. 6.7%, *p* < 0.05).

### 3.4 Trypanocide combination patterns used on farms

Drugs reported to have been used during the past 30 days on farms or, for professionals, that they had administered during the past 30 days are shown in Table 5. In Table 6, these results were used to calculate the number and proportion of farms on which the different drugs were used, or the number and proportion of professionals who reported using these drugs during the past 30 days.

**Table 5:** Drugs administered during the past 30 days (number of cases).

Drugs administered in past 30 days	All farms with ruminants	Farms with both cattle and sheep/goats	Farms with cattle with/without sheep/goats	Farms with sheep/goats but no cattle	Farms with cattle but no sheep/goats	Drug shop attendants and extension officers
DA only	204 (45.2)	90 (47.1)	136 (44.3)	71 (47.3)	43 (39.1)	41 (51.9)
DA + Ab	67 (14.9)	25 (13.1)	52 (16.9)	15 (10.0)	27 (24.5)	0 (0.0)
DA + ISM	46 (10.2)	31 (16.2)	44 (14.3)	2 (1.3)	13 (11.8)	33 (41.8)
DA + ISM + Ab	28 (6.2)	19 (9.9)	27 (8.8)	2 (1.3)	7 (6.4)	1 (1.3)
DA + ISM + HB	0 (0.0)	0 (0.0)	0 (0.0)	0 (0.0)	0 (0.0)	1 (1.3)
DA + ISM + HB + Ab	3 (0.7)	1 (0.5)	1 (0.3)	2 (1.3)	0 (0.0)	0 (0.0)
ISM only	20 (4.4)	8 (4.2)	20 (6.5)	1 (0.7)	11 (10.0)	1 (1.3)
ISM + Ab	1 (0.2)	1 (0.5)	1 (0.3)	0 (0.0)	0 (0.0)	0 (0.0)
Ab only	59 (13.1)	12 (6.3)	19 (6.2)	41 (27.3)	6 (5.5)	0 (0.0)
None	23 (5.1)	4 (2.1)	7 (2.3)	16 (10.7)	3 (2.7)	2 (2.5)
Totals	451 (100)	191 (100)	307 (100)	150 (100)	110 (100)	79 (100)

DA, Diminazene aceturate; ISM, Isometamidium chloride; HB, Homidium bromide; Ab, Antibiotic. Percentages in brackets.

**Table 6:** Total number of cases administering the different types of drug.

Drugs administered in past 30 days	All farms with ruminants	Farms with both cattle and sheep/goats	Farms with cattle with/without sheep/goats	Farms with sheep/goats but no cattle	Farms with cattle but no sheep/goats	Drug shop attendants and extension officers
DA or ISM or HB	369 (81.8)	175 (91.6)	281 (91.5)	93 (62.0)	101 (92.8)	77 (97.5)
DA	348 (77.2)	166 (86.9)	260 (84.7)	92 (61.3)	90 (81.8)	76 (96.2)
ISM	98 (21.7)	60 (31.4)	93 (30.3)	7 (4.7)	31 (28.2)	36 (45.6)
HB	3 (0.7)	1 (0.5)	1 (0.3)	2 (1.3)	0 (0.0)	1 (1.3)
DA + ISM	77 (17.0)	51 (26.7)	72 (23.5)	6 (4.0)	20 (18.2)	35 (44.3)
DA or ISM or HB + Ab	99 (22.0)	46 (24.1)	81 (26.4)	19 (12.7)	34 (30.9)	1 (1.3)
Ab	158 (35.0)	58 (30.4)	100 (32.6)	60 (40.0)	40 (36.4)	1 (1.3)
None	23 (5.1)	4 (2.1)	7 (2.3)	16 (10.7)	3 (2.7)	2 (2.5)
Total cases	451 (100)	191 (100)	307 (100)	150 (100)	110 (100)	79 (100)

Percentages in brackets.

More than four-fifths of farmers (81.8%) reported that one or more type of trypanocidal drug had been used on their farm during the past 30 days (Table 6). All but two (97.5%) professionals reported that they had administered one or more type of trypanocidal drug during the same period; this could have been either to their own livestock (all professionals reported that they also kept ruminant livestock) or as a fee-paying service to animals owned by others. In comparison, around one-third of farmers (35.0%) reported that antibiotics had been used on their farms during this period but just one drug shop attendant or extension officers (1.3%) reported having used an antibiotic during the past 30 days (Table 6).

Diminazene aceturate (DA) was by far the most used trypanocide: 77.2% of farmers reported that DA had been used on their farms in the last 30 days while 21.7% reported the use of isometamidium chloride (ISM), just three (0.7%) reported that homidium bromide (HB) had been used and 5.1% reported no drugs had been used. Among professionals, 96.2% reported that they had used DA, 45.6% reported using ISM and just one reported using HB during the past 30 days (Table 6).

Seventeen percent of farmers and 44.3% of professionals reported that they had used both DA and ISM in the past 30 days. Twenty-two percent of farmers but just one professional reported that they had used both a trypanocide and an antibiotic during the past 30 days (Table 6).

Drug usage varied markedly between farms where cattle were kept and farms where just sheep and goats were kept: 92.8% of farms where cattle were kept reported that a trypanocide had been administered during the past 30 days compared to 62.0% of farms that just kept sheep and goats (Table 6). Diminazene aceturate was the most used drug on both types of farms; 84.7% of farms with cattle reporting using this drug compared to 61.3% of farms with just sheep and goats. Isometamidium chloride usage was much less common on farms with just sheep and goats: 30.3% of farms with cattle used ISM compared to just 4.7% of farms with just sheep and goats. Similarly, far more farms with cattle used both DA and ISM: 23.5% of farms with cattle reported doing so compared to 4.0% of farms with just sheep and goats. All these differences in proportions between farms with/without cattle are statistically significant at *p* < 0.05. Antibiotic usage was slightly higher on farms with just sheep and goats: 36.4% of farms with cattle reported using antibiotics compared to 40.0% of farms with just sheep and goats.

Farmers reported that trypanocidal drugs were administered to animals in their herds or flocks between one and five times a month; on three-quarters of farms this was done once a month. Since the most used trypanocide is DA, which has curative but not prophylactic activity, it is assumed this means on an individual animal basis based on perceived need.

### 3.5 Observance of drug withdrawal periods following treatment

Only 35.9% of farmers and even fewer, 13.8%, of professionals, reported that they observed the recommended withdrawal times following administration of trypanocidal drugs (Table 7). Farmers who kept just cattle were more likely to observe withdrawal times than farmers who just kept sheep/goats (38.2 vs. 24.0%; *p* < 0.05). For professionals, this likely means that they did not personally follow up on the animals once they had been treated. However, less than 5% of all respondents, whether farmers or professionals, knew the recommended withdrawal times for either meat or milk for the most commonly used trypanocide, DA. Drug shop attendants and extension officers demonstrated better theoretical knowledge than farmers on the correct dosage of trypanocidal drug: overall around twice as many of the former knew the correct dose (for DA, 62.0% of professionals compared to 31.0% of farmers; for ISM, 60.8% compared to 33.5%).

**Table 7:** Trypanocide withdrawal practices and dosing.

Trypanocide practices	Farms with both cattle and sheep/goats (*N* = 191)	Farms with sheep/goats but no cattle (*N* = 150)	Farms with cattle but no sheep/goats (*N* = 110)	Farmers (*N* = 451)	Professionals (*N* = 79)	Farms with sheep/goats and cattle only		Farmers vs. Professionals	
						OR (95% CI)	*p* values	OR (95% CI)	*p* values
Trypanocide withdrawals done	84 (44.0)	36 (24.0)	42 (38.2)	162 (35.9)	11 (13.9)	1.9 (1.1–3.3)	0.01	0.3 (0.2–0.6)	<0.001
Correct milk withdrawal period on DA^a^	7 (3.7)	3 (2.0)	4 (3.6)	14 (3.1)	2 (2.5)	1.8 (0.4–10.1)	0.45	0.8 (0.2–3.6)	1.00
Correct meat withdrawal period on DA^b^	13 (6.8)	3 (2.0)	4 (3.6)	20 (4.4)	1 (1.3)	1.8 (0.4–10.1)	0.45	0.3 (0.0–2.1)	0.34
Correct dose to treat an adult cow: DA	24 (12.6)	102 (68.0)	14 (12.7)	140 (31.0)	49 (62.0)	0.1 (0.0–0.1)	<0.0001	3.6 (2.2–6.0)	<0.001
Correct dose to treat an adult cow: ISM	31 (16.2)	101 (67.3)	19 (17.3)	151 (33.5)	48 (60.8)	0.1 (0.1–0.2)	<0.0001	3.1 (1.9–5.0)	<0.001
Correct dose to treat an adult cow: HB	92 (48.2)	114 (76.0)	73 (66.4)	279 (61.9)	56 (70.9)	0.6 (0.3–1.1)	0.09	1.5 (0.9–2.5)	0.13

DA, Diminazene aceturate; ISM, Isometamidium chloride; HB, Homidium bromide. DA withdrawal period for milk a = 3 days, b = 20 days for meat.

### 3.6 Trypanocide dosage and prophylactic practices

On average, the professionals identified close to the correct number of sachets or tablets of DA, ISM or HB needed to treat a hypothetical 400 kg bovine at the recommended dose while farmers suggested around double the recommended dose of DA or ISM was needed (Table 8). The choice of hypothetical 400 kg was based on information on cattle live weights in the study area provided by professional informants during the prequestionnaire trial. When farmers who kept only cattle were compared with those who only kept sheep/goats, the former suggested on average three-times the recommended dose of DA and more than twice the recommended dose of ISM, while the latter suggested close to the recommended dose in each case.

**Table 8:** Estimates of number of sachets/tablets of trypanocides needed to treat a 400 kg bovine.

Trypanocide	Recommended dose rate (mg/kg/bodyweight)	Number of sachets/tablets per 400 kg bovine for recommended dose	Farms with both cattle and sheep/goats	Farms with sheep/goats but no cattle	Farms with cattle but no sheep/goats	Farmers	Professionals
			Mean (Median, IQR)				
Diminazene aceturate	3.5 mg/kg	1.3 sachets^1^	3.6 (4.0, 3)^c^	1.8 (1.0, 1)^b^	3.9 (5.0, 2)^c^	3.1 (3.0, 4)^c^	1.6 (1.0, 1)^a^
Isometamidium chloride	0.5 mg/kg	1.6 sachets^2^	3.7 (4.0, 3)^c^	1.9 (1.0,2)^a^	3.8 (4.0, 2)^c^	3.1 (4.0, 4)^c^	1.6 (1.0, 1)^#^
Homidium bromide	1 mg/kg	1.6 tablets^3^	2.0 (2.0, 2)^b^	1.5 (1.0, 0)^#^	1.7 (1.0, 1)^#^	1.8 (1.0, 1)^a^	1.5 (1.0, 1)^#^

One sample *t*-test statistic. ^a^*p* value < 0.05, ^b^*p* < 0.001, and ^c^*p* < 0.0001. ^#^*p* > 0.05. ^1^Standard sachet contain 1.05 g active ingredient. ^2^Standard sachet contains 125 mg active ingredient. ^3^Tablet contains 250 mg active ingredient.

In addition to using trypanocides, 69.8% (315/451) of farmers also reported using a topical veterinary pesticide for the control of ticks and tsetse, despite only one in five reporting that they were aware that tsetse flies were involved in the transmission of AAT.

Farmers reported spending a median expenditure of USD 103 (mean USD 213.6) each month on trypanocides (range USD 2.2–USD 6504) on their farms, although the range was very large, depending on the farm size (USD 2.2–USD 6504). As a proportion of the monthly income from livestock sales, farmers reported a median expenditure of 6.9% (mean = 12.2%) on trypanocidal drugs on their farms (range from 0 to close to 100%). Both the proportion of income and expenditure were higher on farms that kept cattle than those that did not.

## 4 Discussion

The current study found that on around two-thirds of farms, trypanocidal drugs were being administered by farmers and that almost all drugs were obtained from privately-owned drug shops. Best practice for controlling trypanosomiasis in cattle, sheep, and goats, was not being followed since prescription-only medicines were being routinely administered by farmers without professional supervision and in the absence of a definitive diagnosis.

The most used trypanocide in cattle, sheep, and goats was diminazene aceturate (DA). This drug is cheaper and more widely available than isometamidium chloride (ISM) in the study area (personal observation), and less likely to cause a local reaction at the site of injection (Giordani et al., 2016).

In this study, the way in which farmers administered trypanocidal drugs was compared to the way drug shop attendants (unqualified assistants and diploma-holding technicians) and extension officers administered these products. While most drug shop attendants and all extension officers were better educated than farmers, and a far greater proportion had received specific training on use of trypanocidal drugs, there was surprisingly, relatively little difference in their ability to use these drugs appropriately and according to the manufacturer’s instructions.

There was no evidence that knowledge of trypanosomiasis epidemiology influenced the use of therapeutics since farmers lack access to routine laboratory analysis for pathogen speciation. Compared to drug shop attendants and extension workers, farmers were more likely to use only DA to treat trypanosomiasis; more likely to use antibiotics as well as trypanocides; and they estimated, on average, that twice the recommended dose of DA and ISM was needed to treat a hypothetical 400 kg bovine. There are several possible reasons for this. Firstly, cattle weights can be problematic to assess correctly (Machila et al., 2008), in this study we used a hypothetical weight of 400 kg bovine, Ankole crosses generally have a mean weight of 476 kg (Manzi et al., 2018) while Zebu cattle from central and western Uganda range from 150 to 340 kg (Kagoro-Rugunda et al., 2018) due to genetic, dietary, husbandry practices and geographical location differences. Cattle weight estimations have been found to be difficult not only for farmers but also for clinicians in Kenya demonstrating the need to interpret perceived animal weight estimates presented here with caution (Machila et al., 2008). The differences between dead weight (killing out and slaughterhouse deductions) and liveweight (purchase weight) and resulting price differentials at the market can also result in confusion.

Secondly, during the widely publicized, large-scale Stamp Out Sleeping Sickness Campaign (SOS), to eliminate zoonotic *Trypanosoma brucei rhodesiense,* the causative agent of Human African Trypanosomiasis (HAT) from the cattle zoonotic reservoir in Uganda, a dose of 7 mg of DA per kg bodyweight, was used (Roderick et al., 2000). This is double the dose recommended to treat the most pathogenic AAT species of trypanosome in cattle (*Trypanosoma congolense* and *Trypanosoma vivax*) as a higher dose is needed to eliminate *Trypanosoma brucei* s.l. as recommended by the manufacturer.^1^ Although the current study area was not in the target area for SOS it appears that messages about the benefits of this approach may have spread beyond the SOS area (Welburn and Coleman, 2015; Hamill et al., 2017; Wangoola et al., 2019).

Finally, emergence of trypanocidal drug resistance is commonly ascribed to under-dosing and it is noteworthy that the current study suggests that farmers in this study tended to use more than the recommended dosages of DA and ISM, not less. A similar finding was reported by Roderick et al. (2000) (Wangoola et al., 2019), where Masai pastoralists administered DA and HB to their cattle and tended to give more than the recommended dose. Sub-standard trypanocides with less than the stated amount of active ingredient as well as counterfeit products with no active ingredient have been reported in many African countries (Perry et al., 2005; Bengaly et al., 2018; Tekle et al., 2018) and it is possible that increased dosing may be as a response to either perceived or actual poor quality drugs in the marketplace.

Traditionally, one of the main ways recommended to reduce the risk of trypanocide resistance emerging is the use of the “sanative pair” concept. Diminazene aceturate and ISM are chemically distinct and periodically switching between the two active ingredients is widely considered to be an effective way to prevent drug resistant strains of trypanosomes emerging (Watson, 2013). The current study indicates that only one in every six farmers were using the sanative pair approach (slightly more, around one in five, for those who were cattle keepers), although this practice appeared to be more widely used by drug shop attendants or extension officers. This may be an underestimate, as respondents were only asked about their trypanocide usage during the past 30 days, and it is possible that they switched drugs beyond this timeframe.

The finding that farmers reported using antibiotics much more commonly than drug shop attendants and extension officers does raise concerns about overuse and misuse of this critical class of drugs (Ndaki et al., 2021). From the farmers’ perspective, however, use of both trypanocidal drugs and antibiotics is perhaps a rational response in an environment where tick-borne diseases (Kasozi et al., 2019), many of which can be treated with antibiotics, and trypanosomiasis are both prevalent and definitive diagnosis is not normally available. The very low usage of antibiotics reported by drug shop attendants and extension officers is a surprising finding that warrants further investigation.

The observation that most respondent farmers reported that they used a topical veterinary pesticide to control ticks and tsetse on their animals is encouraging. This approach to controlling tsetse, as part of an integrated approach to controlling trypanosomiasis (Bardosh et al., 2013; Muhanguzi et al., 2014), that is cost effective (Okello et al., 2021) for farmers has been actively promoted in northern Uganda since 2006 by the Stamp-Out Sleeping Sickness (SOS) campaign began (Welburn and Coleman, 2015). That campaign, which also involved field training of veterinary undergraduate students from Makerere University, may have had longer lasting impacts when they took their learning into professional practice in Uganda.

## 5 Conclusion

It is likely that for the foreseeable future, livestock keepers in the cattle belt of southwestern Uganda will continue to treat their own animals using drugs obtained from private drug shops and without the benefit of expert supervision or definitive diagnosis. The reported overdosing with trypanocides and observation that farmers were using a topical veterinary pesticide to control ticks and tsetse on their animals was unexpected outside of the SOS districts. Although the current study area is not within the target area for SOS it appears that messages about the benefits of double dosing of trypanocides for *T. brucei* s.l., and application of topical veterinary pesticides for prevention of re-infection by trypanosomes and, treatment of tick-borne-diseases, may have spread beyond the SOS area (Waiswa et al., 2020) and warrants further investigation.

Some aspects of trypanocidal drug use by farmers would benefit from greater emphasis, support, and training, particularly as regard live weight estimations and drug dosing. A study exploring drug quality in the region would be helpful in gaining a deeper understanding how farmers and practitioners are making decisions on dosing. It is in the best interests of farmers, animal health professionals, drug shops owners, veterinary pharmaceutical companies, state extension services, and the wider local and global community to promote best practice for the use of antimicrobials, and a multi-stakeholder campaign to increase awareness of the sanative pair concept and the importance of following drug withdrawal periods could be a useful way forward. Such approaches could reduce the risk of drug resistant strains of trypanosomes emerging, enhance food safety and support safe use of antimicrobials.

## Data availability statement

The raw data supporting the conclusions of this article will be made available by the authors, without undue reservation.

## Ethics statement

The studies involving humans were approved by Edinburgh Medical School Research Ethics Committee (22-EMREC-022). The studies were conducted in accordance with the local legislation and institutional requirements. Written informed consent for participation in this study was provided by the participants’ legal guardians/next of kin. The animal studies were approved by Ethics Committee at the College of Veterinary Medicine of Makerere University (SVAR-IACUC/114/2022). Written informed consent was obtained from the owners for the participation of their animals in this study.

## Author contributions

KIK: Conceptualization, Data curation, Formal analysis, Investigation, Methodology, Validation, Writing – original draft, Writing – review & editing. ETM: Conceptualization, Data curation, Formal Analysis, Funding acquisition, Investigation, Methodology, Project administration, Resources, Software, Supervision, Validation, Writing – review & editing. KRS: Data curation, Formal Analysis, Methodology, Project administration, Resources, Supervision, Validation, Visualization, Writing – review & editing. SCW: Conceptualization, Data curation, Formal Analysis, Funding acquisition, Investigation, Methodology, Project administration, Resources, Software, Supervision, Validation, Visualization, Writing – review & editing.

## Footnotes

^1^ https://www.ceva-africa.com/en/Products/Product-list/VERIBEN-Rrecommended
