# Supplementary material for: Trypanocide usage in the cattle belt of southwestern Uganda
Source: Front Microbiol. 2023 Dec 13;14:1296522. doi: 10.3389/fmicb.2023.1296522 (PMC10759318; doi:10.3389/fmicb.2023.1296522)
Supplement: Supplementary file 1 [file Data_Sheet_1.docx]

**Trypanocide Usage in the Cattle Belt of Southwestern Uganda**

Keneth Iceland Kasozi^1,2*,^ Ewan Thomas MacLeod^1^, Keith Sones^3^, Susan Christina Welburn^1,4*^

**Affiliations**

1. Infection Medicine, College of Medicine and Veterinary Medicine, Institute for Regeneration and Repair, Edinburgh Bio-Quarter, 4-5 Little France Drive, Edinburgh EH16 4UU, United Kingdom
2. School of Medicine, Kabale University, Box 317 Kabale, Uganda
3. Keith Sones Associates, United Kingdom
4. Zhejiang University-University of Edinburgh Joint Institute, Zhejiang University, International Campus, 718 East Haizhou Road, Haining 314400, China

^*^Correspondence authors: KIK (keneth.kasozi@ed.ac.uk and kicelandy@kab.ac.ug) and SCW (sue.welburn@ed.ac.uk)

**Supplementary file 1: Questionnaire on knowledge and practices on trypanocides**

| Demographics: These are social demographic questions | |
| --- | --- |
| Participant consent form name initials |  |
| Location/Village 1: Drug shop, 2= Farm |  |
| Gender 1 = Male, 2 = Female, 3 = Binary, 4 = Prefer not to say |  |
| Age (years) | We then clustered these as presented in the manuscript |
| Position on the premises: 1 = Head, 2 = Second in-charge, 3 = Family member (volunteer), 4 = employee | This was done across the board |
| Occupation at the premises: 1 = Employee/Herdsman, 2 = Farmer, 3 = extension officer (Animal health only), 3 = Drug shop attendant, 4 = Drug shop technician |  |
| Education level: 1 = No formal education, 2 = Basic education, 3 = Tertiary |  |
| Animals at my premises (select all that apply)  1= Large ruminants (cattle)  2 =Small ruminants (goats and sheep)  3 = small animals (pigs, chickens and rabbits)  4 = Pets (dogs and cats) | During analysis, we only captured those with ruminants to keep the work focused |
| Number of large ruminants (cattle) | We met a great unwillingness in the community to share true values |
| Major breed of cattle:  1 = Exotic (Hostein freisian, Boran, etc)  2 = Bos indicus/Bos taurus or Local  3 = Cross breed  4 = I don’t know |  |
| Small ruminant numbers |  |
| Major breed of small ruminats  1 = Exotic  2 = Local  3 = Crosses  4 = I don’t know |  |
| Number of small animals |  |
| Major breed of pigs and chickens  1=Exotic  2 = Local  3 = Cross  4 = I don’t know |  |
| Number of pets (cats and dogs) that I have |  |
| Major pet breeds at premises  1 = Exotic  2=Local  3 =Cross  4= I don’t know |  |
| Major trypanocide used in the last 30 days (select all that apply)  1 = DA (e.g., veriben)  2 = ISM (e.g., samorin)  3 = Hb (tablets)  4 = Antibiotics  5=None |  |
| Freq of trypanocide usage last 30 days (scale: 1-5) | Scale refers to frequency of application |
| Livestock production system at premises  1 = Dairy  2 = Beef  3=Dual purpose  4=Fattening  5=Subsistence(family) | Subsequently clustered these to semi and commercial |
| Estimated weekly livestock sales (income in UgX) | Converted to USD |
| Estimated monthly livestock sales (income in UgX) | Converted to USD |
| Seasonal (6 month) expenditures on trypanocides (UgX) | Converted to USD |
|  |  |
| Knowledge. (This section will assess your knowledge on trypanocide resistance | |
| Have attended extension trainings on trypanocide drug usage in Uganda  1 =yes  2=no | meetings |
| Best approach for controlling trypanosomiasis 5=1 Rest =0  1=Acaricides  2=Antibiotics  3=Bush burning  4=Ethnomedicine  5=Trypanocides  6=I don’t know | Identify trypanocides (multiple options allowed to identify guessing) |
| Major insects involved in trypanosomiasis 2 =1 Rest = 0  1=Mosquitoes  2=Tsetse flies  3=House flies  4=Frogs  5= I don’t know | Identify tsetse |
| Trypanosomiasis burden is common in our area during the  1=Dry/sunny season  2=Wet/rainy season  3= I don’t know | Identify rainy |
| Practice questions |  |
| Most reliable source of trypanocides  1= Government outlets  2= Private outlets  3 = Black market  4 = I don’t know |  |
| Cheapest source of trypanocides  1= Government outlet  2 = Private outlet  3 = Black market  4 = Price is uniform  5 = I don’t know |  |
| Trypanocide withdrawal days are observed at my premises  1=yes  2=no |  |
| I withdraw milk after treatment with DA for…days |  |
| I withdraw meat after treatment with DA for…days |  |
| Extension officers are accessible in my community  1=yes  2=no |  |
| Extension officers are reliable/available with treatments  1=yes  2=0 |  |
| I  administer trypanocides intravenously (IV)  1=Yes  2=no |  |
| I administer trypanocides intramuscularly (IM)  1=Yes  2=no |  |
| I routinely administer trypanocides with antibiotics  1=yes  2=no |  |
| I use pesticides weekly to control tsetse flies  1=yes  2=no |  |
| I use pesticides on my farm monthly to control ticks  1=yes  2=no |  |
| I use pesticides for both tick and tsetse fly control  1=yes  2=no |  |
| Administration of trypanocides on the farm  1= Farmer  2= Drug shop attendant  3 = Extension officer  4 = I don’t know |  |
| To treat a 400 kg animal, I mix …..sachets of DA  Scale: 1-5 |  |
| To treat a 400 kg animal, I mix …..sachets of ISM  Scale: 1-5 |  |
| To treat a 400 kg animal, I mix …..tablets of Hb  Scale: 1-5 |  |
| Source of water used to mix trypanocides  1= Well  2= Community borehole  3 =Tap  4 = Water stream  5 = Shops  6 = I don’t know |  |
| Amount of money spent on water procured to mix trypanocides to treat (~10) animals |  |
