# Supplementary material for: Trypanocide usage in the cattle belt of southwestern Uganda
Source: Front Microbiol. 2023 Dec 13;14:1296522. doi: 10.3389/fmicb.2023.1296522 (PMC10759318; doi:10.3389/fmicb.2023.1296522)
Supplement: Supplementary file 2 [file Data_Sheet_2.docx]

**Trypanocide Usage in the Cattle Belt of Southwestern Uganda**

**Authors**

Keneth Iceland Kasozi^1,2*,^ Ewan Thomas MacLeod^1^, Keith Sones^3^, Susan Christina Welburn^1,4*^

**Affiliations**

1. Infection Medicine, College of Medicine and Veterinary Medicine, Institute for Regeneration and Repair, Edinburgh Bio-Quarter, 4-5 Little France Drive, Edinburgh EH16 4UU, United Kingdom
2. School of Medicine, Kabale University, Box 317 Kabale, Uganda
3. Keith Sones Associates, United Kingdom
4. Zhejiang University-University of Edinburgh Joint Institute, Zhejiang University, International Campus, 718 East Haizhou Road, Haining 314400, China

^*^Correspondence authors: KIK (keneth.kasozi@ed.ac.uk and kicelandy@kab.ac.ug) and SCW (sue.welburn@ed.ac.uk)

**Supplementary file 2: ISRCTN 54255798 URL**

<https://doi.org/10.1186/ISRCTN54255798>

**Supplementary file 3: R script on analysis**

####Knowledge + Practice on Ruminant farms

#Install packages##

if(!require("pacman"))install.packages("pacman") #if needed

pacman::p_load(pacman, party, rio, tidyverse)

### Importing data###

library(readxl)

df <- read_excel("~/OneDrive - University of Edinburgh/Re_Analysis_July_2023/Re_Analysis_August/TrypKAP_RStudio_Finale_230810.xlsx",

sheet = "Rumaints", na = "0")

str(df)

glimpse(df)

## TRANSFORM THE CHARACTERS TO FACTORS

df$Gender <- as.factor(df$Gender)

df$PositionPremises <- as.factor(df$PositionPremises)

df$Role <- as.factor(df$Role)

df$Educ <- as.factor(df$Educ)

df$AnimalsatPremises <- as.factor(df$AnimalsatPremises)

df$AnimalCat <- as.factor(df$AnimalCat)

df$Cattle <- as.factor(df$Cattle)

df$shoats <- as.factor(df$shoats)

df$Livestock <- as.factor(df$Livestock)

df$CattleBreed <- as.factor(df$CattleBreed)

df$Shoatbreed <- as.factor(df$Shoatbreed)

df$majortrypanocides30days <- as.factor(df$majortrypanocides30days)

df$Drugusepatternmonthly <- as.factor(df$Drugusepatternmonthly)

df$productionSystem <- as.factor(df$productionSystem)

df$productionCAT <- as.factor(df$productionCAT)

df$sourceoftrypanocides <- as.factor(df$sourceoftrypanocides)

df$CattleBreed <- as.factor(df$CattleBreed)

df$AnimalsatPremises <- as.factor(df$AnimalsatPremises)

#knowledge

df$ `I have sufficient knowledge on trypanosomiasis epidemiologyfromEXTENSION` <- as.factor(df$ `I have sufficient knowledge on trypanosomiasis epidemiologyfromEXTENSION`)

df$attendedextensiontrainings <- as.factor(df$attendedextensiontrainings)

df$`Best approach to control trypanososmiasis is using` <- as.factor(df$`Best approach to control trypanososmiasis is using`)

df$`Trypanosomiasis burden is common in our area during the` <- as.factor(df$`Trypanosomiasis burden is common in our area during the`)

df$`Insects involved in trypanosomiasis` <- as.factor(df$`Insects involved in trypanosomiasis`)

#Practice

df$Admin_IV <- as.factor(df$Admin_IV)

df$Admin_IM <- as.factor(df$Admin_IM)

df$`TrypCides+Ab` <- as.factor(df$`TrypCides+Ab`)

df$`Pesticide_vector-Control` <- as.factor(df$`Pesticide_vector-Control`)

df$Pesticide_Ticks_control <- as.factor(df$Pesticide_Ticks_control)

df$Pesticide_Tick_Fly_control <- as.factor(df$Pesticide_Tick_Fly_control)

df$PesticideResistance <- as.factor(df$PesticideResistance)

df$Administration <- as.factor(df$Administration)

df$DA <- as.factor(df$DA)

df$ISM <- as.factor(df$ISM)

df$Hb <- as.factor(df$Hb)

df$waterMix <- as.factor(df$waterMix)

df$Trypanocideffective <- as.factor(df$Trypanocideffective)

df$Resistance <- as.factor(df$Resistance)

df$Diagnosis <- as.factor(df$Diagnosis)

df$Location <- as.factor(df$Diagnosis)

df$Job <- as.factor(df$Job)

df$Extension_Access <- as.factor(df$Extension_Access)

df$Admin_Treatments <- as.factor(df$Admin_Treatments)

df$Cheapestsourceoftrypanocides <- as.factor(df$Cheapestsourceoftrypanocides)

df$Withdrawal <- as.factor(df$Withdrawal)

df$Extension_Trust <- as.factor(df$Extension_Trust)

df$Tryp2District <- as.factor(df$Tryp2District)

df$Tryp2UGA <- as.factor(df$Tryp2UGA)

df$`African trypanosomiasis affects me` <- as.factor(df$`African trypanosomiasis affects me`)

df$WithdrawMeatYES <- as.factor(df$WithdrawMeatYES)

df$WithdrawMilkYES <- as.factor(df$WithdrawMilkYES)

glimpse(df)

str(df)

summary(df$AnimalCat)

summary(df$Livestock)

summary(df)

#Transform age into classes

#bin the continous data and create new variable in dataframe

df$AgeCAT <- cut(df$Age,

breaks = c(14,18,26,35,45,61,82)

)

summary(df$AgeCAT)

###for farmers ==unskilled

##Step 1: General: (TABLE 1=df1/2), df0=farmers/herders

df0 <- filter(df, Job =="Unskilled") %>% #laymen = farmers and herdsmen

as_tibble()

summary(df0)

count(df0)

df00 <- filter(df, Job =="Skilled") %>% #experts =extension officers and drugshop attendants

as_tibble()

summary(df00$AnimalCat)

###SUBSET FOR CATTLE, SHOATS ALONE to compare frequencies with combined with Job

#subset 1 =dfa

df1 <- filter(df0, AnimalCat == "cattle") %>%

as_tibble()

summary(df1)

df2 <-filter(df0, AnimalCat == "shoats") %>%

as_tibble()

summary(df2)

df3 <- filter(df0, AnimalCat == "Ruminants") %>%

as_tibble()

#Table 3 trypanocide usage in farms and experts #Table 4 is used to generate Table 4 through summation of cases for the individual characters

summary(df3$majortrypanocides30days)

summary(df2$majortrypanocides30days)

summary(df1$majortrypanocides30days)

summary(df4$majortrypanocides30days)

summary(df0$majortrypanocides30days)

#Table 5. variables influencing practice #here we repat specific summaries

#df1 =laymen in cattle

#df2 = laymen in shoats

#df3 =laymen in ruminants

#df0 =laymen

#df00 =experts

#Administer treatments

summary(df3$Administration)

summary(df2$Administration)

summary(df1$Administration)

summary(df0$Administration)

summary(df00$Administration)

#Correct route of admin

summary(df3$Admin_IV)

summary(df2$Admin_IV)

summary(df1$Admin_IV)

summary(df0$Admin_IV)

summary(df00$Admin_IV)

summary(df3$Admin_IM)

summary(df2$Admin_IM)

summary(df1$Admin_IM)

summary(df0$Admin_IM)

summary(df00$Admin_IM)

#cheap source of trypanocides

summary(df3$Cheapestsourceoftrypanocides)

summary(df2$Cheapestsourceoftrypanocides)

summary(df1$Cheapestsourceoftrypanocides)

summary(df0$Cheapestsourceoftrypanocides)

summary(df00$Cheapestsourceoftrypanocides)

#cheap source of drugs

summary(df3$sourceoftrypanocides)

summary(df2$sourceoftrypanocides)

summary(df1$sourceoftrypanocides)

summary(df0$sourceoftrypanocides)

summary(df00$sourceoftrypanocides)

#water source

summary(df3$waterMix)

summary(df2$waterMix)

summary(df1$waterMix)

summary(df0$waterMix)

summary(df00$waterMix)

#Table 6. trypanocide usage practices

summary(df3$Withdrawal)

summary(df2$Withdrawal)

summary(df1$Withdrawal)

summary(df0$Withdrawal)

summary(df00$Withdrawal)

#correct milk withdrawal on DA

summary(df3$WithdrawMilkYES)

summary(df2$WithdrawMilkYES)

summary(df1$WithdrawMilkYES)

summary(df0$WithdrawMilkYES)

summary(df00$WithdrawMilkYES)

#correct meat withdrawal on DA

summary(df3$WithdrawMeatYES)

summary(df2$WithdrawMeatYES)

summary(df1$WithdrawMeatYES)

summary(df0$WithdrawMeatYES)

summary(df00$WithdrawMeatYES)

#correct dose of DA

summary(df3$DA)

summary(df2$DA)

summary(df1$DA)

summary(df0$DA)

summary(df00$DA)

#correct ISM dose

summary(df3$ISM)

summary(df2$ISM)

summary(df1$ISM)

summary(df0$ISM)

summary(df00$ISM)

#correct Hb dose

summary(df3$Hb)

summary(df2$Hb)

summary(df1$Hb)

summary(df0$Hb)

summary(df00$Hb)

##Table 7. estimated number of sachets/tablets

#DA

summary(df3$`DA sachets treat 400kg animal`)

summary(df2$`DA sachets treat 400kg animal`)

summary(df1$`DA sachets treat 400kg animal`)

summary(df0$`DA sachets treat 400kg animal`)

summary(df00$`DA sachets treat 400kg animal`)

#ISM

summary(df3$ISMsachetstreat400kg)

summary(df2$ISMsachetstreat400kg)

summary(df1$ISMsachetstreat400kg)

summary(df0$ISMsachetstreat400kg)

summary(df00$ISMsachetstreat400kg)

#Hb

summary(df3$`Hb sachetstreat400kg animal`)

summary(df2$`Hb sachetstreat400kg animal`)

summary(df1$`Hb sachetstreat400kg animal`)

summary(df0$`Hb sachetstreat400kg animal`)

summary(df00$`Hb sachetstreat400kg animal`)

##extra checks

summary(df00$Gender)

summary(df00$Age)

summary(df00$CattleNumbers)

summary(df00$Shoats)

#Calculate odds ratios

install.packages('epitools')

library(epitools)

#create OD matrix

Information <- c('farmer', 'professional')

freq <- c('correct', 'fail')

ODinform <- matrix(c(218, 242, 67, 13), nrow = 2, ncol = 2, byrow = TRUE)

dimnames(ODinform) <- list('farmer'=Information, 'professional'=freq)

##Odds ratio, risk ratios for computations on univariates on Knowledge

df4 <- table(df$Job, df$`I have sufficient knowledge on trypanosomiasis epidemiologyfromEXTENSION`)

df4 <- cbind(df4[,2], df4[,1])

df4

barplot(df4, beside = T, legend =T)

install.packages('epiR')

library(epiR)

epi.2by2(df4, method = "cohort.count", conf.level = 0.95)

##multiple subseting for Table 2

df5 <- table(df$Job, df$`Best approach to control trypanososmiasis is using`)

program <- c('professional', 'farmer')

outcome <- c('Correct', 'Wrong')

df5 <- matrix(c(54, 25, 290, 161), nrow=2, ncol=2, byrow=T)

dimnames(df5) <- list('Job'=program, 'trypanocides'=outcome)

oddsratio(df5)

df6 <- table(df$Job, df$`Insects involved in trypanosomiasis`)

df6 <- matrix(c(54, 25, 99, 352), nrow=2, ncol=2, byrow=T)

dimnames(df6) <- list('Job'=program, 'insects'=outcome)

oddsratio(df6)

df7 <- table(df$Job, df$`Trypanosomiasis burden is common in our area during the`)

df7 <- matrix(c(69, 10, 356, 95), nrow=2, ncol=2, byrow=T)

dimnames(df7) <- list('Job'=program, 'rainy season'=outcome)

oddsratio(df7)

#Admin treatments Table 5 on major variables influencing practice

df8 <- table(df$Job, df$Administration)

df8 <- matrix(c(11, 68, 55, 396), nrow=2, ncol=2, byrow=T)

dimnames(df8) <- list('Job'=program, 'Drugshop attendants'=outcome)

oddsratio(df8)

df88 <- matrix(c(8,102, 34, 116), nrow=2, ncol=2, byrow=T)

dimnames(df88) <- list('Job'=program, 'Drugshop attendants in laymen'=outcome)

oddsratio(df88)

df8a <- matrix(c(16, 63, 98, 353), nrow=2, ncol=2, byrow=T)

dimnames(df8a) <- list('Job'=program, 'Extension officer'=outcome)

oddsratio(df8a)

df8aa <- matrix(c(29,81, 25, 125), nrow=2, ncol=2, byrow=T)

dimnames(df8aa) <- list('Job'=program, 'extension officer in laymen'=outcome)

oddsratio(df8aa)

df8b <- matrix(c(52, 27, 298, 153), nrow=2, ncol=2, byrow=T)

dimnames(df8b) <- list('Job'=program, 'Farmer'=outcome)

oddsratio(df8b)

df8bb <- matrix(c(73, 37, 91, 59), nrow=2, ncol=2, byrow=T)

dimnames(df8bb) <- list('Job'=program, 'Farmer in laymen'=outcome)

oddsratio(df8bb)

df9 <- table(df$Job, df$Admin_IV)

df9

barplot(df9, beside = T, legend =T)

epi.2by2(df9, method = "cohort.count", conf.level = 0.95)

df9a <- matrix(c(78, 1, 443, 8), nrow = 2, ncol = 2, byrow=T)

dimnames(df9a) <- list('Job'=program, 'IV route in laymen'=outcome)

oddsratio(df9a)

df10 <- table(df$Job, df$Admin_IM)

df10 <- cbind(df10[,2], df10[,1])

df10

barplot(df9, beside = T, legend =T)

epi.2by2(df10, method = "cohort.count", conf.level = 0.95)

df10a <- matrix(c(106, 4, 143, 7), nrow = 2, ncol = 2, byrow=T)

dimnames(df10a) <- list('Job'=program, 'IM route in laymen'=outcome)

oddsratio(df10a)

df11 <- matrix(c(61, 18, 329, 122), nrow=2, ncol=2, byrow=T)

df11

dimnames(df11) <- list('Job'=program, 'Private outlets'=outcome)

oddsratio(df11)

df11a <- matrix(c(85, 25, 103, 47), nrow=2, ncol=2, byrow=T)

df11a

dimnames(df11a) <- list('Job'=program, 'Private outlets in laymen'=outcome)

oddsratio(df11a)

#df12 <- matrix(c(0, 79, 2, 449), nrow=2, ncol=2, byrow=T)

#dimnames(df12) <- list('Job'=program, 'Black market'=outcome)

#df12

#oddsratio(df12)

#df12a <- see medcalc

df13 <- matrix(c(3, 76, 4, 447), nrow=2, ncol=2, byrow=T)

dimnames(df13) <- list('Job'=program, 'Government outlet'=outcome)

df13

oddsratio(df13)

epi.2by2(df13, method = "cohort.count", conf.level = 0.95)

df14 <- matrix(c(80, 0, 446, 4), nrow=2, ncol=2, byrow=T)

df14

dimnames(df14) <- list('Job'=program, 'Private source'=outcome)

oddsratio(df14)

df15 <- matrix(c(0, 80, 5, 455), nrow=2, ncol=2, byrow=T)

df15

dimnames(df15) <- list('Job'=program, 'Governmetn source'=outcome)

oddsratio(df15)

df15a <- matrix(c(2, 108, 1, 149), nrow=2, ncol=2, byrow=T)

df15a

dimnames(df15a) <- list('Job'=program, 'Governmetn source in laymen'=outcome)

oddsratio(df15a)

df15b <- matrix(c(14, 65, 62, 409), nrow = 2, ncol = 2, byrow = T)

df15b

dimnames(df15b) <- list('Job'=program, 'Uniform price'=outcome)

oddsratio(df15b)

df15b1 <- matrix(c(12, 98, 13, 137), nrow = 2, ncol = 2, byrow = T)

df15b1

dimnames(df15b1) <- list('Job'=program, 'Uniform price in laymen'=outcome)

oddsratio(df15b1)

df15b2 <- matrix(c(1, 78, 54, 397), nrow = 2, ncol = 2, byrow = T)

df15b2

dimnames(df15b2) <- list('Job'=program, 'not sure'=outcome)

oddsratio(df15b2)

df15b3 <- matrix(c(11, 99, 33, 117), nrow = 2, ncol = 2, byrow = T)

df15b3

dimnames(df15b3) <- list('Job'=program, 'not sure in laymen'=outcome)

oddsratio(df15b3)

df15c <- matrix(c(107, 3, 145, 5), nrow = 2, ncol = 2, byrow = T)

df15c

dimnames(df15c) <- list('Job'=program, 'private source of cheap drugs in laymen'=outcome)

oddsratio(df15c)

df15d <- matrix(c(3, 147, 1, 149), nrow = 2, ncol = 2, byrow = T)

df15d

dimnames(df15d) <- list('Job'=program, 'Government source of cheap drugs in laymen'=outcome)

oddsratio(df15d)

#water source

df16 <- matrix(c(4, 75, 28, 423), nrow=2, ncol=2, byrow=T)

dimnames(df16) <- list('Job'=program, 'Borehole'=outcome)

df16

oddsratio(df16)

df16a <- matrix(c(8, 102, 5, 145), nrow=2, ncol=2, byrow=T)

dimnames(df16a) <- list('Job'=program, 'Borehole in laymen'=outcome)

df16a

oddsratio(df16a)

df17 <- matrix(c(10, 69, 67, 384), nrow=2, ncol=2, byrow=T)

dimnames(df17) <- list('Job'=program, 'Bottled water'=outcome)

df17

oddsratio(df17)

df17a <- matrix(c(21, 89, 10, 140), nrow=2, ncol=2, byrow=T)

dimnames(df17a) <- list('Job'=program, 'Bottled water in laymen'=outcome)

df17a

oddsratio(df17a)

df18 <- matrix(c(34, 45, 159, 292), nrow=2, ncol=2, byrow=T)

dimnames(df18) <- list('Job'=program, 'Tap'=outcome)

df18

oddsratio(df18)

df18a <- matrix(c(45, 65, 61, 89), nrow=2, ncol=2, byrow=T)

dimnames(df18a) <- list('Job'=program, 'Tap in laymen'=outcome)

df18a

oddsratio(df18a)

df19 <- matrix(c(9, 70, 190, 261), nrow=2, ncol=2, byrow=T)

dimnames(df19) <- list('Job'=program, 'Well'=outcome)

df19

oddsratio(df19)

df20 <- matrix(c(35, 75, 70, 80), nrow=2, ncol=2, byrow=T)

dimnames(df20) <- list('Job'=program, 'Well in laymen'=outcome)

df20

oddsratio(df20)

df21 <- matrix(c(22, 57, 4, 447), nrow=2, ncol=2, byrow=T)

dimnames(df21) <- list('Job'=program, 'I dont remember'=outcome)

df21

oddsratio(df21)

###Odds ratios on Table 6

df22 <- table(df$Job, df$Withdrawal)

df22 <- cbind(df22[,2], df22[,1])

df22

epi.2by2(df22, method = "cohort.count", conf.level = 0.95)

df22a <- matrix(c(42, 68, 36, 114), nrow=2, ncol=2, byrow=T)

dimnames(df22a) <- list('Job'=program, 'withdrawal in laymen'=outcome)

df22a

oddsratio(df22a)

df23 <- table(df$Job, df$DA) #correct dose of DA administered for adult

summary(df2$DA)

df23 <- cbind(df23[,2], df23[,1])

df23

epi.2by2(df23, method = "cohort.count", conf.level = 0.95)

df23a <- matrix(c(14, 96, 102, 48), nrow=2, ncol=2, byrow=T)

dimnames(df23a) <- list('Job'=program, 'DA in laymen'=outcome)

df23a

oddsratio(df23a)

df24 <- table(df$Job, df$ISM) #correct dose of ISM administered for adult

df24

df24 <- cbind(df24[,2], df24[,1])

df24

epi.2by2(df24, method = "cohort.count", conf.level = 0.95)

df24a <- matrix(c(19, 91, 101, 49), nrow=2, ncol=2, byrow=T)

dimnames(df24a) <- list('Job'=program, 'ISM in laymen'=outcome)

df24a

oddsratio(df24a)

df25 <- table(df$Job, df$Hb) #correct dose of Hb administered for adult

df25

df25 <- cbind(df25[,2], df25[,1])

df25

epi.2by2(df25, method = "cohort.count", conf.level = 0.95)

df25a <- matrix(c(73,37, 114, 36), nrow=2, ncol=2, byrow=T)

dimnames(df25a) <- list('Job'=program, 'Hb in laymen'=outcome)

df25a

oddsratio(df25a)

df26 <- table(df$Job, df$WithdrawMilkYES) #correct withdrawal period for milk against DA

df26

df26 <- cbind(df26[,2], df26[,1])

df26

epi.2by2(df26, method = "cohort.count", conf.level = 0.95)

df26a <- matrix(c(4, 106, 3, 147), nrow=2, ncol=2, byrow=T)

dimnames(df26a) <- list('Job'=program, 'DA withdrawal in milk in laymen'=outcome)

df26a

oddsratio(df26a)

df27 <- table(df$Job, df$WithdrawMeatYES) #correct withdrawal period for meat against DA

df27

df27 <- cbind(df27[,2], df27[,1])

df27

epi.2by2(df27, method = "cohort.count", conf.level = 0.95)

df27a <- matrix(c(4, 106, 3, 147), nrow=2, ncol=2, byrow=T)

dimnames(df27a) <- list('Job'=program, 'DA withdrawal in meat in laymen'=outcome)

df27a

oddsratio(df27a)

#####OR for Table 3

#construct matrixes for all for k by 2

library(epiR)

library(epitools)

df28 <- matrix(c(41, 38, 204, 247), nrow=2, ncol=2, byrow=T)

dimnames(df28) <- list('Job'=program, 'DA only'=outcome)

df28

oddsratio(df28)

df28a <- matrix(c(43, 67, 71, 79), nrow=2, ncol=2, byrow=T)

dimnames(df28a) <- list('Job'=program, 'DA only in laymen'=outcome)

df28a

oddsratio(df28a)

df29 <- matrix(c(27, 83, 15, 135), nrow=2, ncol=2, byrow=T)

dimnames(df29) <- list('Job'=program, 'DA+Ab in laymen'=outcome)

df29

oddsratio(df29)

df30 <- matrix(c(33, 46, 46, 405), nrow=2, ncol=2, byrow=T)

dimnames(df30) <- list('Job'=program, 'DA+ISM'=outcome)

df30

oddsratio(df30)

df30a <- matrix(c(13,97, 2, 148), nrow=2, ncol=2, byrow=T)

dimnames(df30a) <- list('Job'=program, 'DA+ISM in laymen'=outcome)

df30a

oddsratio(df30a)

df31 <- matrix(c(41, 109, 6, 104), nrow=2, ncol=2, byrow=T)

dimnames(df31) <- list('Job'=program, 'Ab only in laymen'=outcome)

df31

oddsratio(df31)

df32 <- matrix(c(2, 77, 23, 428), nrow=2, ncol=2, byrow=T)

dimnames(df32) <- list('Job'=program, 'No treatment'=outcome)

df32

oddsratio(df32)

df32a <- matrix(c(16, 134, 3, 107), nrow=2, ncol=2, byrow=T)

dimnames(df32a) <- list('Job'=program, 'No treatment in laymen'=outcome)

df32a

oddsratio(df32a)

df33 <- matrix(c(1, 78, 28, 423), nrow=2, ncol=2, byrow=T)

dimnames(df33) <- list('Job'=program, 'DA+ISM+Ab'=outcome)

df33

oddsratio(df33)

df33a <- matrix(c(7,103, 2, 148), nrow=2, ncol=2, byrow=T)

dimnames(df33a) <- list('Job'=program, 'DA+ISM+Ab in laymen'=outcome)

df33a

oddsratio(df33a)

df34 <- matrix(c(1, 78, 20, 431), nrow=2, ncol=2, byrow=T)

dimnames(df34) <- list('Job'=program, 'ISM only'=outcome)

df34

oddsratio(df34)

df34a <- matrix(c(11, 99, 1, 149), nrow=2, ncol=2, byrow=T)

dimnames(df34a) <- list('Job'=program, 'ISM only in laymen'=outcome)

df34a

oddsratio(df34a)

####################Keiths additional analysis###############

df35 <- matrix(c(77, 151, 369, 672), nrow=2, ncol=2, byrow=T)

dimnames(df35) <- list('Job'=program, 'DA/ISM/Hb'=outcome)

df35

oddsratio(df35)

df35a <- matrix(c(101, 184, 93, 183), nrow=2, ncol=2, byrow=T)

dimnames(df35a) <- list('Job'=program, 'DA/ISM/Hb in laymen'=outcome)

df35a

oddsratio(df35a)

df36 <- matrix(c(76, 152, 348, 693), nrow=2, ncol=2, byrow=T)

dimnames(df36) <- list('Job'=program, 'DA'=outcome)

df36

oddsratio(df36)

df36a <- matrix(c(90, 195, 92, 184), nrow=2, ncol=2, byrow=T)

dimnames(df36a) <- list('Job'=program, 'DA in laymen'=outcome)

df36a

oddsratio(df36a)

df37 <- matrix(c(36, 192, 98, 943), nrow=2, ncol=2, byrow=T)

dimnames(df37) <- list('Job'=program, 'ISM'=outcome)

df37

oddsratio(df37)

df37a <- matrix(c(31, 254, 7, 269), nrow=2, ncol=2, byrow=T)

dimnames(df37a) <- list('Job'=program, 'ISM in laymen'=outcome)

df37a

oddsratio(df37a)

df38 <- matrix(c(1, 227, 3, 1038), nrow=2, ncol=2, byrow=T)

dimnames(df38) <- list('Job'=program, 'Hb'=outcome)

df38

oddsratio(df38)

df39 <- matrix(c(35,193, 77, 964), nrow=2, ncol=2, byrow=T)

dimnames(df39) <- list('Job'=program, 'DA+ISM'=outcome)

df39

oddsratio(df39)

df39a <- matrix(c(20, 265, 6, 270), nrow=2, ncol=2, byrow=T)

dimnames(df39a) <- list('Job'=program, 'DA+ISM in laymen'=outcome)

df39a

oddsratio(df39a)

df40 <- matrix(c(123, 918, 1, 227), nrow=2, ncol=2, byrow=T)

dimnames(df40) <- list('Job'=program, 'Ab'=outcome)

df40

oddsratio(df40)

df40a <- matrix(c(60, 216, 40, 245), nrow=2, ncol=2, byrow=T)

dimnames(df40a) <- list('Job'=program, 'Ab in laymen'=outcome)

df40a

oddsratio(df40a)

df41 <- matrix(c(2, 226, 23, 1018), nrow=2, ncol=2, byrow=T)

dimnames(df41) <- list('Job'=program, 'No drugs/none'=outcome)

df41

oddsratio(df41)

df41a <- matrix(c(16, 260, 3, 282), nrow=2, ncol=2, byrow=T)

dimnames(df41a) <- list('Job'=program, 'No drugs/none in laymen'=outcome)

df41a

oddsratio(df41a)

######more data out########

summary(df0$Extension_Access)

summary(df00$Extension_Access)

summary(df3$Extension_Access)

summary(df2$Extension_Access)

summary(df1$Extension_Access)

summary(df3$Extension_Trust)

summary(df2$Extension_Trust)

summary(df1$Extension_Trust)

summary(df0$Extension_Trust)

summary(df00$Extension_Trust)

#work with the original dataframe since it has the different livestock dfs

Access1 <- table(df$Job, df$Extension_Access)

Access1

Access1 <- cbind(Access1[,2], Access1[,1])

Access1

epi.2by2(Access1, method = "cohort.count", conf.level = 0.95)

Acc2 <- matrix(c(78, 72, 72, 38), nrow=2, ncol=2, byrow=T)

dimnames(Acc2) <- list('Job'=program, 'EO access in farmers'=outcome)

Acc2

oddsratio(Acc2)

Reliab1 <- table(df$Job, df$Extension_Trust)

Reliab1

Reliab1 <- cbind(Reliab1[,2], Reliab1[,1])

Reliab1

oddsratio(Reliab1)

Reliab2 <- matrix(c(68, 82, 47, 63), nrow=2, ncol=2, byrow=T)

dimnames(Reliab2) <- list('Job'=program, 'EO reliability in farmers'=outcome)

Reliab2

oddsratio(Reliab2)

summary(df0$Cattle)

table(df0$majortrypanocides30days, df0$Cattle)

###ditional analysis####

summary(df$Exp_Income_Monthly)

summary(df0$Exp_Income_Monthly)

summary(df0$MontlyincomeUSD)

#########mean dosage

?std.error()

std.error(df$Age)

install.packages('plotrix')

library(plotrix)

#calculate std error for one sample t-test

#shorter path

library(ggpubr)

install.packages('rstatix') # provides pipe-friendly R functions for easy statistical analyses

t.test(df3$`DA sachets treat 400kg animal`, mu = 1.3) #Ruminants on DA

t.test(df2$`DA sachets treat 400kg animal`, mu = 1.3) #shoats on DA

t.test(df1$`DA sachets treat 400kg animal`, mu = 1.3)

t.test(df0$`DA sachets treat 400kg animal`, mu = 1.3)

t.test(df00$`DA sachets treat 400kg animal`, mu = 1.3)

#one sample t-test for ISM

t.test(df3$ISMsachetstreat400kg, mu = 1.6)

t.test(df2$ISMsachetstreat400kg, mu = 1.6)

t.test(df1$ISMsachetstreat400kg, mu = 1.6)

t.test(df0$ISMsachetstreat400kg, mu = 1.6)

t.test(df00$ISMsachetstreat400kg, mu = 1.6)

#One sample t-test for Hb

t.test(df3$`Hb sachetstreat400kg animal`, mu = 1.6)

t.test(df2$`Hb sachetstreat400kg animal`, mu = 1.6)

t.test(df1$`Hb sachetstreat400kg animal`, mu = 1.6)

t.test(df0$`Hb sachetstreat400kg animal`, mu = 1.6)

t.test(df00$`Hb sachetstreat400kg animal`, mu = 1.6)

####begin again

#describe the economic trend on the farms

summary(df3$MontlyincomeUSD, df3$MonthlyexpendituresTrypanocidesUSD) #combined. Not useful

summary(df3$Exp_Income_Monthly)

# correlations

dc <- df %>%

select(MontlyincomeUSD, MonthlyexpendituresTrypanocidesUSD, Exp_Income_Monthly) %>%

as_tibble() %>%

print()

dc[is.na(dc)] <- 0

names(dc) <- c("Inc", "Exp", "%Exp_Inc_Ratio")

dc

#correlation of dc variables

dc %>%

cor() %>%

round(1) %>%

print()

cor.test(dc$Inc, dc$Exp)

cor.test(dc$Inc, dc$`%Exp_Inc_Ratio`)

cor.test(dc$Exp, dc$`%Exp_Inc_Ratio`)

###Economic loss using regression models

Econ1 <- df %>%

select(Job, AnimalCat, Livestock, MontlyincomeUSD, MonthlyexpendituresTrypanocidesUSD, Exp_Income_Monthly) %>%

as_tibble() %>%

print()

names(Econ1) <- c("Job", "AnimalCat", "Livestock", "Income", "Expenses", "EIR" )

Econ1

Econ1 %>%

ggplot(aes( x = Expenses, y = Income))+

geom_point()+

geom_smooth(method = "lm")+

labs(title = "Income and Expenses",

x = "Expenses",

y = "Income")

model1 <- lm(Income~Expenses, data = Econ1)

model1

require(ggplot2)

levels(Econ1$AnimalCat)

library(dplyr)

levels(Econ1$AnimalCat) <- c("Cattle", "Both", "Shoats")

levels(Econ1$AnimalCat)

levels(Econ1$Job)

levels(Econ1$Job) <- c("Experts", "Laymen")

levels(Econ1$Job)

names(Econ1)[names(Econ1) == 'Job'] <- 'Participant'

summary(Econ1)

Econ1 %>%

ggplot(aes(x = AnimalCat, fill = Participant)) +

geom_bar() +

xlab("Livestock species") +

ylab("Frequency") +

theme_classic() +

theme(legend.position = "right") +

theme(axis.text.x = element_text(angle = 15, hjust = 1)) +

theme(text = element_text(size = 12)) +

theme(axis.text.y = element_text(size = 14)) +

theme(axis.text.x = element_text(size = 12)) +

theme(axis.title.y = element_text(size = 14))

#description of econonics

Econ1 %>%

group_by(AnimalCat) %>%

summarise( n =n(),

mean = mean(Income),

median_inc = median(Income),

iqr_inc = IQR(Income),

min_inc = min(Income),

max_inc = max(Income)

) # on income descriptions

Econ1 %>%

group_by(AnimalCat) %>%

summarise( n =n(),

mean = mean(Expenses),

median_inc = median(Expenses),

iqr_inc = IQR(Expenses),

min_inc = min(Expenses),

max_inc = max(Expenses) # on expense descriptions

)

Econ1 %>%

group_by(AnimalCat) %>%

summarise( n =n(),

mean = mean(EIR),

median_inc = median(EIR),

iqr_inc = IQR(EIR),

min_inc = min(EIR),

max_inc = max(EIR) # business health status

)

#################################################################Income and livestock numbers

###Economic loss using regression models

library(tidyverse)

Econ2 <- df %>%

select(Job, AnimalCat, Livestock, CattleNumbers, Shoats, MontlyincomeUSD, MonthlyexpendituresTrypanocidesUSD, Exp_Income_Monthly) %>%

as_tibble() %>%

print()

names(Econ2) <- c("Job", "AnimalCat", "Livestock", "Cattle", "Shoats", "Income", "Expenses", "EIR" )

Econ2

Econ2 %>%

ggplot(aes( x = Cattle, y = Income, fill= Job))+

geom_point()+

geom_smooth(method = "lm")+

labs(title = "Income and cattle numbers",

x = "Cattle numbers",

y = "Income")

model2 <- lm(Income~Cattle, data = Econ2)

model2

require(ggplot2)

levels(Econ1$AnimalCat)

library(dplyr)

levels(Econ1$AnimalCat) <- c("Cattle", "Both", "Shoats")

levels(Econ1$AnimalCat)

levels(Econ1$Job)

levels(Econ1$Job) <- c("Experts", "Laymen")

levels(Econ1$Job)

names(Econ1)[names(Econ1) == 'Job'] <- 'Participant'

summary(Econ1)

Econ1 %>%

ggplot(aes(x = AnimalCat, fill = Participant)) +

geom_bar() +

xlab("Livestock species") +

ylab("Frequency") +

theme_classic() +

theme(legend.position = "right") +

theme(axis.text.x = element_text(angle = 15, hjust = 1)) +

theme(text = element_text(size = 12)) +

theme(axis.text.y = element_text(size = 14)) +

theme(axis.text.x = element_text(size = 12)) +

theme(axis.title.y = element_text(size = 14))

#description of econonics

Econ2 %>%

group_by(AnimalCat) %>%

summarise( n =n(),

mean = mean(Income),

median_inc = median(Income),

iqr_inc = IQR(Income),

min_inc = min(Income),

max_inc = max(Income)

) # on income descriptions

Econ1 %>%

group_by(AnimalCat) %>%

summarise( n =n(),

mean = mean(Expenses),

median_inc = median(Expenses),

iqr_inc = IQR(Expenses),

min_inc = min(Expenses),

max_inc = max(Expenses) # on expense descriptions

)

Econ1 %>%

group_by(AnimalCat) %>%

summarise( n =n(),

mean = mean(EIR),

median_inc = median(EIR),

iqr_inc = IQR(EIR),

min_inc = min(EIR),

max_inc = max(EIR) # business health status

)

#graphical presentation of the same results above on income

#Boxplots

plot1 <- (Econ1 %>%

ggplot(aes(x = AnimalCat, y = Income, color = Participant))+

geom_boxplot()+

labs(title = "Monthly income",

x = "Livestock on farms",

y = "Income in USD"))

plot2 <- (Econ1 %>%

ggplot(aes(x = AnimalCat, y = Expenses, color = Participant))+

geom_boxplot()+

labs(title = "Monthly expenses",

x = "Livestock on farms",

y = "Expenses in USD"))

plot3 <- (Econ1 %>%

ggplot(aes(x = AnimalCat, y = EIR, color = Participant))+

geom_boxplot()+

labs(title = "Financial assessment",

x = "Livestock on farms",

y = "Percentage EIR score"))

plot1 + theme_bw() + theme(panel.border = element_blank(), panel.grid.major = element_blank(),

panel.grid.minor = element_blank(), axis.line = element_line(colour = "black"))

plot2 + theme_bw() + theme(panel.border = element_blank(), panel.grid.major = element_blank(),

panel.grid.minor = element_blank(), axis.line = element_line(colour = "black"))

plot3 + theme_bw() + theme(panel.border = element_blank(), panel.grid.major = element_blank(),

panel.grid.minor = element_blank(), axis.line = element_line(colour = "black"))

#################################################################################################################################################
